# Barriers and facilitators for isoniazid preventive therapy (IPT) administration in children under 5 years of age in the Dominican Republic

**DOI:** 10.1186/s12879-022-07333-2

**Published:** 2022-04-11

**Authors:** Grey Idalia Benoit Vásquez, Ana Lucia Morrobel, Dione Benjumea-Bedoya, Helena del Corral-Londoño

**Affiliations:** 1 Administradora de Estancias Infantiles Salud Segura, Santo Domingo, Dominican Republic; 2 National Directorate of Epidemiology (DIGEPI), Ministry of Public Health, Santo Domingo, Dominican Republic; 3 Grupo de Epidemiología, Facultad Nacional de Salud Pública, Universidad de Antioquia, Medellín, Colombia; 4 Programa de Control de Tuberculosis, Ministerio de Salud Pública, Santo Domingo, Dominican Republic; 5 Grupo de investigación MICROBA, Escuela de Microbiología, Universidad de Antioquia, Medellín, Colombia; 6 Grupo de Investigación en Salud Familiar y Comunitaria. Facultad de Ciencias de la Salud, Corporación Universitaria Remington, Medellín, Colombia

**Keywords:** Isoniazid preventive therapy, Tuberculosis, Barriers, Facilitators, Person centred approach

## Abstract

**Introduction:**

Throughout the world tuberculosis (TB) is the second leading cause of death due to an infectious agent. The World Health Organization promotes Isoniazid Preventive Therapy (IPT) in children under 5 years who are contacts of persons diagnosed with smear-positive pulmonary TB (SPPTB). In 2019, 33% of children identified as contacts received IPT globally, while in the Americas 11 countries reached coverages ≥ 75%, only 35% did so in the Dominican Republic (DR). The aim of this study was to identify barriers and facilitators for IPT administration in children under 5 in the Area IV Directorate of Health of the DR’s National District.

**Methods:**

Descriptive study, using mixed methods and sequential explanatory approach. We characterized children under 5 years who were contacts of a person with SPPTB. Later, semi-structured interviews and content analysis allowed identification of barriers and facilitators for IPT administration in children who were contacts of a person diagnosed with SPPTB, as perceived by relatives and health system personnel.

**Results:**

A total of 238 children were identified as contacts of 174 persons with SPPTB: 36% of these received IPT while no data on IPT administration was found for 11% of them. The proportion of children who had a tuberculin skin test (TST) done was < 20%. However, those who had the test done had a greater opportunity to receive IPT (OR: 8.12, CI 95%: 1.60–41.35). Barriers identified include socioeconomic conditions of children and families, stigma, lack of information in clinical and follow-up records, lack of coordination between public and private providers and lack of coherence within national regulations. Facilitators include home based care of persons with TB and their contacts, transfer of treatment to a health centre near household, isoniazid availability, provision of information by health-workers and economic support for food and transportation.

**Conclusions:**

Incomplete data, lack of use of TST to rule out active TB, socioeconomic and cultural conditions, were barriers for IPT administration. Implementation of a person centred approach to care was found to be the main facilitator for IPT uptake. Administration of IPT depends predominantly on modifiable health system factors. This allows rapid identification of strategies to improve IPT administration.

## Introduction

Tuberculosis (TB) is still an important global health issue as it accounts for disease in around 10 million people each year. It is one of the 10 leading causes of death around the world and during the last 5 years it has been the leading cause of death from a single infectious agent [1]. In 2019, about 1.04 million children with a new episode of TB were diagnosed and notified, while 15% died from TB and 8986 were diagnosed with multidrug- or rifampicin-resistant TB (MDR/RR-TB) [2].

Paediatric TB represents approximately 6.9% of all new persons diagnosed with smear-positive TB (SPTB) [1] and is present mostly in countries with high burdens of disease. Risk factors for childhood TB are: (a) being a household or close contact of a person with pulmonary TB, b) being under 5 years of age, (c) having HIV infection, (d) having severe malnutrition and (e) having extrapulmonary disease [3].

In the Dominican Republic (DR) TB is a national priority. According to the Pan American Health Organization (PAHO), this country achieved the Millennium Development Goals (MDGs) related to TB prevalence and mortality ahead of time after showing descending incidence [4]. Overall TB incidence decreased from 57.6/100,000 to 40.1 between 2004 and 2014 while pulmonary TB decreased from 40.4/100,000 to 36.0 [5]. However, poverty, inequality, food insecurity, negative effects of human migrations and complex emergencies are among the 5 factors that still need to be addressed in Latin America [6, 7].

Preventing new Mycobacterium tuberculosis (M.tb) infections and their progression to active disease, as well as reducing the burden of disease and death caused by TB are fundamental for its eradication [1]. For this reason, the World Health Organization (WHO) promotes the use of Isoniazid Preventive Therapy (IPT) as a simple and effective method to prevent M.tb reactivation [8–11].

According to PAHO-WHO guidelines, symptoms of TB in children include weight loss or failure to thrive, fever and cough; it is also important to consider the history of contact with a person with infectious TB disease [10, 12]. Children with these symptoms need to be assessed for TB. In those cases where active TB is ruled out and they are contacts of persons with smear-positive pulmonary TB (SPPTB), IPT should be administered for 6–9 months in spite of Bacillus of Calmette-Guerin (BCG) vaccination [8, 10, 11, 13, 14]. Worldwide the number of household contacts provided with TB preventive treatment has been small, although they are increasing (423,607 in 2018 and 538,396 in 2019) [15].

In 2019, throughout the world, 1.3 million children under 5 who were contacts of persons with SPPTB were eligible to receive IPT, however only 433,196 (equivalent to 33%) children under 5 years old were provided with TB preventive treatment. This represents an increase from the 349,796 children who received IPT in 2018 and a large increase from 87,242 who did so in 2015. The Americas and the European Region were WHO’s regions which had the highest coverage of preventive treatment for contacts. In the Americas, 11 countries reached coverages of ≥ 75% [15].

PAHO points out that in countries with low TB incidences, as is the case of the DR, there is a need to improve the initiation, termination and reporting of IPT [1].

The DR’s National Guidelines for diagnosis, treatment and prevention of TB in children indicate that, in order to rule out active disease, Stegen and Toledo criteria need to be considered; thus taking into consideration clinical assessment, tuberculin skin testing test (TST), images and history of contact with a person with smear-positive pulmonary tuberculosis (SPPTB) [16].

In the DR, by 2014 35% of children who were contacts of persons with SPPTB received IPT, while 58% completed the treatment [17]. Hence, health authorities established that this coverage gap needs to be narrowed down through an increase from 61 to 90% in children under 5 years of age. This requires knowledge of the factors that influence IPT success [4]. Thus, the objective of this study was to identify barriers and facilitators for the administration of IPT in children under 5 years of age who were in the TB Control Program (TCP) at the IV Area Directorate of Health (DAS-IV) of the National District in the Dominican Republic.

## Population and methods

### Design

A descriptive study, using mixed methods with a sequential explanatory approach was performed in 2017. The latter is described as a design that occurs in two distinct interactive phases in order to use the initial quantitative results to guide the collection of qualitative information and thus explain the phenomenon more clearly and precisely [18, 19]. First, in the quantitative phase, we characterized children under 5 years of age who had been in contact with a person diagnosed with SPPTB, using clinical, socioeconomic, and demographic information. Then, we identified barriers and facilitators for the administration of IPT in children contacts of persons with SPPTB by exploring perceptions of relatives and other actors involved.

### Setting

The study was performed at DAS-IV in Santo Domingo National District in the DR. This corresponds to an area with the greatest population density (29,024 inhabitants per km^2^) and smallest extension (14.2 km^2^), which in 2015 accounted for 50% of confirmed cases in the province [20]. In 2016, incidence of pulmonary TB in the DAS-IV (54.7/100,000 inhabitants) was similar to that for the province (54.3/100,000 inhabitants) and almost twice that of the country (36.4/100,000 inhabitants) [20]. The DAS-IV presents the highest rates of overcrowding in Santo Domingo, with the worst sanitary conditions: deficient garbage collection services, irregular availability of drinking water, presence of sewage and poor rainwater drainage. Household crowding conditions were extracted from index cases’ follow-up forms and clinical records.

### Participants

**Fig. 1:**
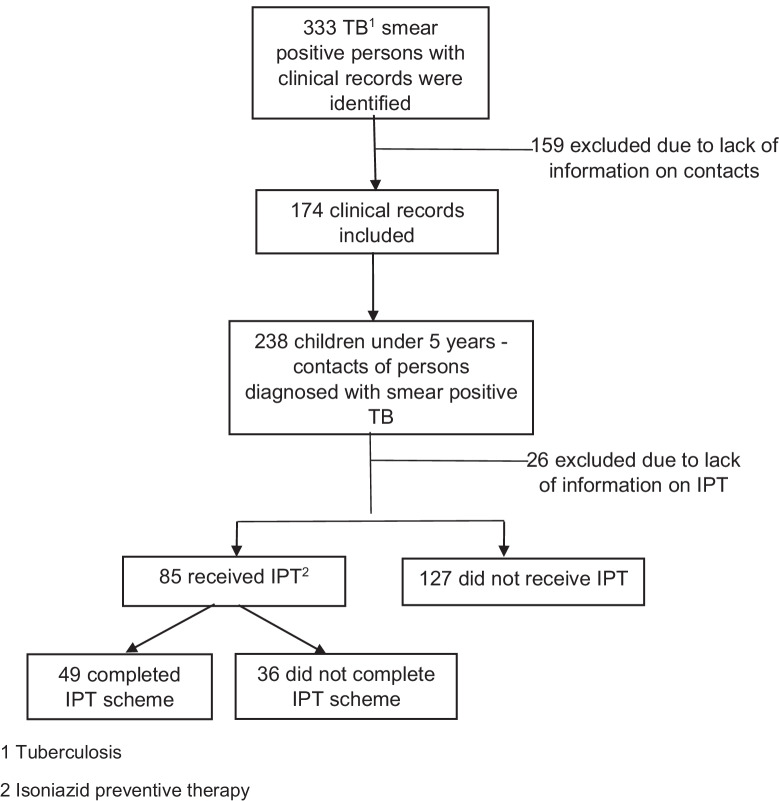
Selection of children under 5 who were contacts of a person diagnosed with smear-positive TB. 1. Tuberculosis. 2. Isoniazid preventive therapy

The quantitative stage considered all children who had been identified as contacts of persons diagnosed with smear-positive TB between January 1, 2015, and December 31, 2016 through the Information System of the TCP. For the qualitative stage, 11 institutions and 13 relatives were selected. One relative refused to continue participating into the study during the interview. Information on contacts was obtained from the records on the person with SPPTB (Fig. 1).

### Quantitative stage

Demographic, clinical, and sociodemographic characteristics of the child under 5 years of age as well as it’s index case, were collected retrospectively. Based on current regulations, a household contact was defined as a child who resides in the same household as a patient and a community contact is a child who lives in the same neighborhood, visits, is frequently visited by or holds a close relationship with a person with TB. Those who received IPT were identified by the information in the follow-up form which was found in the nearest healthcare centre. Those cases in which the child received less than 80% of the treatment regimen were considered as receiving incomplete IPT and those that received 80% or more ITP were considered to have completed treatment [21].

### Qualitative stage

Considering information on location, neighborhood zone and reception of IPT resulting from the analysis of the quantitative data, relatives of children under 5 years of age who were contacts of persons diagnosed with smear-positive TB and health system personnel, were selected to participate in semi-structured interviews to identify barriers and facilitators for the administration of IPT.

Selection of health system personnel (program coordinator, zone managers, health centre program managers and health promoters) was done using purposive sampling. This was done taking into consideration at least one of the following criteria: health zones with highest percentages of children identified as contacts of a person diagnosed with smear-positive TB, a healthcare centre with the highest number of children who initiated and completed IPT, a healthcare centre with the highest number of children who initiated but didn’t complete IPT and a healthcare centre with the greatest number of children who failed to receive IPT. For the selection of relatives, they had to be legal guardians of the child and live-in neighborhoods with the greatest number children who did not receive IPT (4 relatives); residing in those neighborhoods with the highest numbers of children who completed IPT (4 relatives); and from the neighborhoods with the greater number of children who failed to complete IPT (4 relatives). To put forward different arguments that influence the reception of IPT, the study considered: relationship of index case with the child, type of contact, crowding and socioeconomic level of the child. Interviews were performed after obtaining agreement to participate.

Barriers were defined as all activities, situations and/or actions that impeded delivery or reception of isoniazid and IPT completion [21, 22]. Facilitators were defined as all actions, activities and/or situations that allowed for the delivery, reception and completion of IPT [21].

### Data collection

The information was collected sequentially. Between April 17th and May 12th of 2017, quantitative data was collected from secondary sources such as: medical records of persons with TB, first home visit forms and follow up forms. Visits were made to the health centres where the files of those children who were identified as contacts of persons with SPPTB in 2015 and 2016 were located. Each patient’s follow up form includes information on weekly IPT offer and refusal which is recorded using checkboxes.

Then, from July 3rd to August 3rd of 2017, semi-structured interviews with the institutional actors involved in the administration of IPT and with the families of children who were identified in 2015 and 2016 as contacts of persons with SPPTB, were audio recorded and conducted by the first author with a witness present. Institutional actors were visited at their workplace, and the children’s relatives at their homes. Interviews had a maximum duration of 90 min.

A pilot test was carried out in the DAS-IV from April 3rd to 7th, 2017. To validate the instruments, information was extracted by the main researcher and staff from four forms used by the TCP in 33 health centres. Then, two interviews were conducted with health personnel from the NTCP and two interviews with relatives of children under 5 years of age in the localities where the fewest cases occurred.

One relative refused to continue participating in the study during the interview.

### Analysis of information

First, univariate analysis of qualitative variables was performed using absolute and relative frequencies. Normality of the quantitative variables was evaluated using the Kolmogorov–Smirnov test. Quantitative variables that did not meet the normality criteria were transformed. The Chi square test of independence was used to explore associations between dichotomous variables. Fisher's exact test was used for small cell numbers [23]. An exploratory bivariate analysis was performed using logistic regression to summarize the findings and identify possible factors associated with the receipt of IPT. To this end, receipt of IPT was the outcome and being a child contact of a SPPTB case was the exposure considered. Also, adjustment for index case related correlations was performed. Stata 14 IC Software was used.

For the qualitative phase, a study-specific alphanumeric code was assigned to each semi-structured interview to guarantee confidentiality. These were transcribed verbatim, and content analysis was performed. According to Ulin, context analysis can be understood as “A research technique designed to formulate, based on certain data, reproducible and valid inferences applicable to its context” [24]. Such analyses were performed and developed as indicated by Krippendorff, in agreement with previously identified categories, leaving room for emerging categories [25], followed by open coding to identify previous categories in the text, then axial coding to identify a key phenomenon and link categories to subcategories. Finally, selective coding was used to achieve the integration and substantiation of hypotheses that would explain the findings. The transcriptions were made by staff. Transcription’s reviews and coding of interviews were done by the first author. Atlas-Ti Vs 7 software was used.

All the interviews’ transcriptions were returned to the TCP and results were presented to 23 health centres, 8 zone coordinators, 10 health care centre directors as well as the DAS-IV director and TCP coordinator.

## Results

A total of 333 smear-positive case files were reviewed, of which 159 were excluded as they did not have information on contacts of the case. In the remaining 174 files, 238 contacts under 5 years of age were identified (children/case ratio 1.4:1), 212 of them had information about IPT receipt. The median age was 2.7 years with interquartile range 1.0 to 5.0. While 35.7% of children had received IPT, the administration status was unknown for 10.9% of them (see Fig. 1).

Out of 212 of children enrolled, 97% were household contacts, 34.1% had been tested for TST, 47.1% were living in underprivileged neighborhoods from zone 1 nearby the Ozama river and 56.5% lived in overcrowded conditions. Fifteen percent of children [38] were 5 years of age and were included in the study because implementation of IPT offer and administration was found to include them. Similarly, 5.6% [12] children who came from outside the DAS IV area were included, because they came to the study area for TB related care. Differences were found regarding sex, age, and TST application, when the characteristics of children who received and failed to receive IPT were compared (see Table 1).

**Table 1:** Comparison of clinical, socioeconomic, and demographic characteristics of children under 5 who were contacts of a person diagnosed with smear-positive TB regarding their Isoniazid Preventive Therapy (IPT) reception status

Characteristics	IPT recipient n = 85 (%)	Total (n = 212)	OR^b^ (CI 95%)
Sex			
Male	50 (58.8)	104	
Female	35 (41.2)	108	0.50 (0.18–1.37)
Age			
Under 1 year-old	11 (12.9)	25	
1–4 years old	68 (80.0)	149	1.75 (0.48–6.41)
5 years old	6 (7.1)	38	0.40 (0.07–2.42)
No data	0 (0.0)	2	
Contact type			
Community	2 (2.3)	11	
Household	83 (97.7)	200	1
No data	0 (0.0)	1	
Neighborhood by zone^a^			
Neighborhood Zone 1	40 (47.1)	89	
Neighborhood Zone 2	19 (22.4)	53	0.85 (0.25–2.85)
Neighborhood Zone 3	4 (4.7)	17	0.13 (0.02–0.67)
Neighborhood Zone 4	17 (20.0)	41	2.17 (0.1434.79)
Outside DAS-IV area	5 (5.9)	12	
Kinship of index case			
1st degree of consanguinity (parents/brothers/sisters)	53 (62.4)	119	1.69 (0.57–5.08)
2nd degree of consanguinity (grandparents/uncle/aunt)	32 (37.6)	86	1
Other degree (great-grandparents/stepfather)	0 (0.0)	5	
No data	0 (0.0)	2	
Resistance status of index case			
No	74 (87.1)	190	
Yes	11 (12.9)	21	2.33 (0.35–15.27)
No data	0 (0.0)	1	
Specializated health centre attention			
No	66 (77.7)	174	
Yes	19 (22.3)	38	0.40 (0.07–2.36)
Evidence of TST			
No	36 (42.4)	114	
Yes	29 (34.1)	35	8.12 (1.60–41.35)
No data	20 (23.5)	63	
Evidence of BCG vaccination			
No	2 (2.4)	11	
Yes	78 (91.8)	170	2.95 (0.32–27.17)
No data	5 (5.9)	31	
Overcrowding			
No	32 (37.6)	88	
Yes	48 (56.5)	111	2.07 (0.68–6.26)
No data	5 (5.9)	13	

Information System, Program for the Control of Tuberculosis, IV Area Directorate of Health, National District, Dominican Republic

*BCG* Bacillus Calmette-Guérin, *IPT* isoniazid preventive therapy, *TST* tuberculin skin test

^a^Division of territory of the IV Area Directorate of Health of National District by quadrants, limited by Av. Duarte and Av. Padre Castellanos-V Centenario. Neighborhoods: Barrio Zona 1: near to Ozama river (Domingo Savio, María Auxiliadora, La Ciénaga, Mejoramiento Social, Villa Consuelo, Villa Francisca), Barrio Zona 2 (Capotillo, Simón Bolívar, Ensanche Espaillat, Ensanche Luperón, Gualey, 24 de Abril) Barrio Zona 3 (Villa Juana, Ensanche La Fe), Barrio Zona 4 (Cristo Rey, La Zurza, Villas Agrícolas).

^b^OR are adjusted to account for index case related correlations.

### Characteristics of participants

A total of 11 health system employees participated in the interviews. These were: the general manager of the TCP, the coordinator of zone managers, 4 zone managers, 3 managers of the program at healthcare centres, and 2 health promoters with working experience of 7 and 32 years (see Table 2).

**Table 2:** Characteristics of health system personnel participating in interviews

Gender	Time of service in the National Health System (years)	Work role	Professional profile
Female	9	Program nurse in primary care unit	Nursing assistant
Female	12	Program manager in primary care unit	Professional nurse
Female	21	Program manager in primary care unit	Professional nurse
Female	7	Zone managers coordinator	General physician
Female	7	Health promoter	Middle level complete^a^
Female	8	Zone managers	Professional nurse
Female	20	Zone managers	General physician
Male	6	Health promoter	Middle level complete^a^
Female	12	Health promoter	Family and Community Doctor
Female	32	Program coordinator	Public Health master’s degree
Female	10	Zone manager	General physician

^a^Educational level in accordance with the Organic Law of Education of the Dominican Republic no. 66–97 dated April 9, 1997, Article 31: “The Dominican educational system comprises the levels: Initial, Basic, Middle and Higher”

The following relatives were included: 7 parents, 2 uncles and 3 grandmothers. The average age was 32 years (SD 7), 75% of those interviewed were persons diagnosed with SPPTB cases, 92% were household contacts and 58% were female (see Table 3).

**Table 3:** Characteristics of relatives participating in interviews

Gender	Age (years)	Diagnosed with smear-positive TB	Relationship with child	Contact type	Treatment outcome
Female	32	Yes	Mother	Household	IPT not accepted
Female	30	Yes	Mother	Household	IPT scheme complete
Female	37	Yes	Grandmother	Household	IPT scheme complete
Male	31	Yes	Father	Household	IPT scheme incomplete
Male	33	Yes	Father	Household	IPT scheme incomplete
Male	27	Yes	Uncle	Community	IPT not accepted
Male	28	No	Father	Household	IPT scheme complete
Male	29	Yes	Uncle	Household	IPT not accepted
Female	23	No	Mother	Household	IPT not accepted
Female	45	Yes	Grandmother	Household	IPT scheme incomplete
Female^a^	65	Yes	Grandmother	Community	IPT scheme incomplete
Female	30	Yes	Mother	Household	IPT scheme incomplete
Female	48	No	Grandmother	Household	IPT scheme complete

^a^Interview ruled out due to a family member's decision not to continue with it

### Barriers and facilitators

**Fig. 2:**
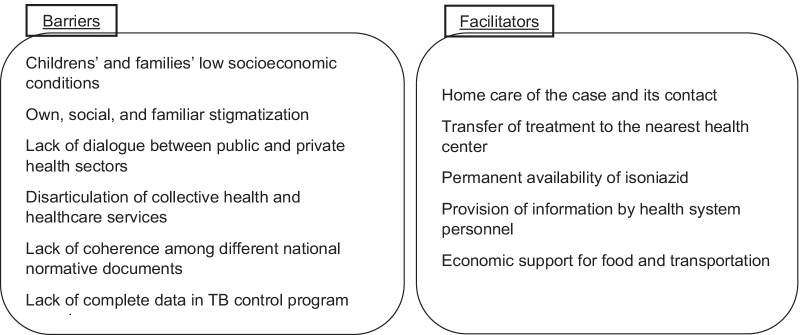
Barriers and facilitators for IPT administration identified

Knowledge-related, health system, behavioral and socioeconomic barriers and facilitators were identified (see Fig. 2).

### Knowledge-related barriers

For health system personnel it is not clear which specialist should do the clinical evaluation to rule out TB. This confusion derives from changes in norms and protocols which indicate that a paediatrician should evaluate the child. Parents and guardians acknowledge different aspects of the disease in terms of routes of contagion and preventive measures; however, they refer not knowing the consequences of incomplete therapy.

### Knowledge-related facilitators

Health system personnel know which is the target population for IPT administration as well as the procedure to identify children who were contacts of a person diagnosed with SPPTB and the need for a clinical evaluation by trained personnel, as well as images, chest X-rays and laboratory tests to rule out TB in children. Furthermore, they know that in case of negative results, IPT needs to be initiated. They are also aware that if one of the tests is positive, the child needs to be entered into the program as an active case. Similarly, they know the indicated therapy’s length and doses, how the medicine should be administered, how information should be recorded in the registration and follow-up forms for a person diagnosed with smear-positive TB, and the importance that relatives and guardians know this information for treatment acceptance and success. Personnel conduct orientation talks with relatives to promote knowledge on how to administer the medicine and explain the process, including its length.

“Follow-up has to be a conjoint effort between us (health system personnel) and parents because they have to collect the medicine or take the child to receive treatment; and we explain how to administer it. We enter the date of admission and when treatment started, including age and weight of the baby. Depending on the weight we administer the medicine” (AI-05).

For health system personnel, supplying information to family and guardians is of outmost importance for IPT acceptance. When health system personnel guide parents and/or guardians, information delivery is effective and accepted more easily.

### Health system related barriers

Frequent changes in health care personnel were found to be a barrier as evidenced by the fact that most missing data from the follow up forms resulted from lack of knowledge of 2 persons who had recently started working in the TB control program.

As explained by health system personnel, decentralization of health services has led to a division in the TCP. Decentralization of services and the lack of articulation between public and private sectors are some of the problems for administering IPT. Sometimes when the private sector rules out active disease, parents hear that preventive treatment is not necessary as they are not ill, hence foregoing the epidemiological criteria as contact cases, inducing parents to doubt TCP guidelines and refuse preventive treatment.

“My children had a TST, and an X-ray done that came back normal. I took them to their pediatrician, who said it was not necessary to use preventive therapy. I found it weird that the pediatrician did not want to prescribe the therapy because I have seen and known of patients who have had children in IPT. I was filled with doubt. As he said not to, and the children were fine, I decided against it” (FT-01).

Health system personnel express that there are flaws in investigating contacts and healthcare centres which do not do house calls even when participating in the program. Community contacts are often forgotten when asking about household contacts of persons diagnosed with smear-positive TB, and even though they do not sleep under the same roof as the person diagnosed with smear-positive TB they spend the day closely and can arrive to the program as a person with SPTB. Five percent [11] of children who were eligible to receive IPT were community contacts of the index case and only 2 of these received preventive therapy.

Sometimes TST is performed at a specialized health care centre which is far from the patient’s home. In addition, there is often a shortage of reagents to perform the skin test in regular health centres, so they must go to specialized ones. This becomes a barrier for IPT administration as relatives tend not attend those specialized centres for testing. Herein we observed that having TST done is associated with increased chances of IPT reception (OR: 8.12, 95% CI 1.60–41.35). Furthermore, some doctors do not prescribe the test to rule out active disease.

The lack of a comprehensive approach for patient and contacts leads to relatives and/or guardians to avoid taking the child for assessment, while the absence of expert psychological and nutritional assessments leads to incomplete and delayed diagnoses.

### Health system related facilitators

According to health system personnel, Isoniazid is available in all healthcare centres. In the case stock runs low, health system personnel notify the area manager to restock. There are also no problems with the provision of supplies for smear microscopies and for the care of children. Another facilitator is the existence of a unit for patients with multidrug-resistant TB and for the care of persons diagnosed with TB who are suspected of some degree of resistance to first and second line antituberculous drugs.

The identification of contacts and cases is done by an active search for respiratory symptoms or by an initial household visit. Furthermore, two other visits take place during the 6 months of preventive treatment. Data from the visits, such as information on contacts under the age of five, are recorded in the registration and follow-up files of the person diagnosed with smear-positive TB, avoiding duplication of records within the program, and ensuring the recording of delivery of isoniazid to parents. Because the length of IPT is equal to or less than that of treatment for the person with active TB, preventive therapy takes place at the same time as that of the person diagnosed with smear-positive TB, making a weekly delivery to avoid additional transportation expenses.

Transfer of preventive treatment delivery for children and anti-tuberculosis treatment for the index case to their homes, school, nursery, or nearest healthcare centre, is a facilitator to families. Both health system personnel and relatives indicate that therapy is facilitated by having a healthcare centre where both the adult index case and their contacts under the age of five can be assisted.

Persons diagnosed with SPPTB, and their contacts receive treatment follow-up from health system personnel, from before the beginning of, during and after the treatment. A good relationship between relatives and health system personnel during follow-up constitute important facilitators as families that receive good treatment towards them, making them feel as equals without stigma, became more receptive to initiating therapy and motivated to taking the persons to periodic assessments and completing IPT. Health system personnel explain:

“If personal rapport is not friendly the person will start to back off. I mean someone ill with TB has to be treated as if they are not sick, give them trust” (AI-07).

As reported by parents and/or guardians, both absence of adverse effects during therapy as well as knowing others who have undergone treatment without adverse events encourages them to administer the medicine to their children.

### Behavioral and socioeconomic barriers

As expressed by health system personnel, the main behavioral barrier is the parents’ or guardians’ decision not to initiate IPT treatment, despite being sensitized by health system personnel and understanding what TB is and the implications of not receiving IPT.

Parents' decision not to administer IPT is influenced by not seeing the disease immediately in the children or by hiding their own disease by omitting to mention the name of TB in the home and surroundings, despite knowing about how contagion takes place, the risks and the effort made by health system personnel.

“They [health promoters] also explained to me that children needed a medicine to prevent from getting the disease, but the mother did not want to administer it” (FT-09).

According to both health system personnel and relatives, the separation of parents entails custody agreements. In many cases, a person who suffers from TB does not get custody, and after moving to a new neighborhood, the child who had begun therapy cannot continue it. Another reason for the separation and decision of where children should reside is the illness suffered by one of the parents, causing the other partner, due to stigmatization, to leave the home taking the children with him or her; or in other situations leave the child with the smear-positive parent, who can sometimes present some disabilities that do not allow them to attend healthcare centres.

The lack of money to reach the healthcare centres and feed the children, as well as living in poor and marginalized neighborhoods constitute social barriers. Family members express that many times the healthcare centres are not close to their homes, therefore going to get the weekly treatment implies spending money they do not have. In addition, 75 of 159 persons (47.2%) diagnosed with SPPTB, who were contacts of a child were found to live in overcrowded conditions.

### Behavioral and socioeconomic facilitators

Acceptance of therapy by parents is the first behavioral facilitator found. After talking with the health system personnel, the decisions of these parents and/or guardians are mainly motivated by preventing children from becoming sick.

“I gave the medicine [to my girl] so she does not get the illness [TB] my son has, because the nurses explained to me there was a risk if I did not give it to her” (FT-13).

Another of the reasons for which children start preventive treatment is having heard, seen, or known someone from the community who has gone through this process. It is in the child’s best interest that health system personnel tell them the truth.

From the health system personnel standpoint, the main facilitator is taking the medicine to persons with TB and contacts when they miss the appointment at the healthcare centre. Another facilitator identified was good rapport with health system personnel at the centre where persons with TB and contacts receive attention.

## Discussion

Children under 5 years of age who are contacts of a person diagnosed with SPPTB are at high risk of infection and disease progression, hence investigation of contacts is essential to diagnose additional cases and prevent vulnerable people from transitioning from latent infection to active disease [1, 10, 27–30].

This research allows to document that, for the Santo Domingo context, around 48% of index cases did not have information on contacts. A study of 2014 in India reported a proportion of data loss of contacts (33.3%) lower than the one for this study [26]. Thus, lack of data is clearly a barrier to administer IPT to contacts. Information on missing data was not found in studies from the American region.

The proportion of children under 5 years of age who received IPT in DAS-IV was 35%, like the proportion registered for the country. This shows that IPT coverage is still lacking in the Dominican territory. A study in Medellin, Colombia reported a proportion of 19.4% of children under 5 years of age receiving IPT [27]. Proportions reported in India are between 26 and 33% in children under 6 years of age [26, 28]; Indonesia [29], Zimbabwe [30] and Ethiopia [31] reported between 50 and 64%; while other studies performed in Benin [32] and Gambia [33] reported proportions greater than 80%.

The proportion of children tested using TST was less than 20%, and only 34% of those who received IPT were tested with TST. This happened in spite of it being one of Stegen and Toledo’s criteria included in national guidelines to rule out active disease and start IPT [16]. Although the opportunity to receive IPT was 8 times higher in those tested than in those who were not, there are few healthcare centres equipped for testing and these are specialized ones. In addition, doctors do not usually prescribe TST to rule out active disease, thus becoming a barrier of the health system for the adequate study and management of contacts. Other studies have reported TST in contacts in proportions greater than 90% [34] and found that the odds of receiving IPT is 40 times higher in those with TST [27].

The treatment cascade for latent TB has been described [35] and, according to this, the critical points we found broken were: getting testing (15.6%), accepting IPT (35.7%) and completing treatment (57.6%). There are some differences between our findings and those of Alsdurf et al. because in the DR IPT was offered to children that had not been tested. However, the final proportion of children that completed treatment was similar in both settings.

Most contacts were found to reside in vulnerable high-risk neighborhood zones, close to highly contaminated rivers, and almost a half live in overcrowded conditions where families do not perceive TB as a risk. Some studies found that treatment drop out is related to household socioeconomic conditions [36], and is 4 times more common among those with lower income [34].

Social marginalization is internally perceived by relatives of children, which leads to self-stigmatization and perception of social stigma by the family and social environment; aggravated by stigmatization from health care providers. These conditions influence parents to refuse IPT, hence becoming an important and modifiable barrier for this intervention. A study found that health system personnel identified stigmatization associated with TB as a key barrier to schedule household visits as it limited their capacity to collect sufficient information to perform proper household follow ups [21]. It is hence necessary to facilitate social support for both patients and contacts [22].

The supply of IPT in everyday practice differs in some aspects from the guidelines for the attention of children and adolescents and the protocol for diagnosis and treatment of TB in the country. These include: the specialist in charge of assessment, house calls, testing to rule out active cases, among others. Similarly, disarticulation between the private sector and national TCP represent a barrier for IPT administration. Studies show that reception of IPT is influenced by the doctor-patient relationship, and decision-makers are making an inefficient effort to guarantee its success [22].

The availability of isoniazid in all healthcare centres, the transfer of treatment to the closest healthcare centre, and, in some cases, taking IPT to the household, are some of the strategies implemented by the TCP which constitute important facilitators. Some countries with high burden of morbidity refer the lack of isoniazid as a barrier for IPT [28, 30, 37, 38]. Personalized healthcare services provided by health promotors facilitate the administration of IPT [21], which together with TB community-based interventions result in better acceptance of the treatment [38].

While programs must meet the goals and purpose of putting an end to TB there are indirect costs not covered by the programs, such as transportation and food. Whether it is the transportation of persons with TB to collect medicines or of health system personnel to administer treatment, the cost must be covered by themselves directly. These strategies are not part of the current guidelines. However, transportation and food costs are a barrier for relatives and/or guardians and children to access health services. It is modifiable. Different authors have reported the absence of visits by health system personnel as a barrier for IPT [21, 29, 39, 40]. However, access to IPT whether at a nearby healthcare centre [41] or in the household, posterior to the acceptance of treatment, constitute a facilitator due to the economic discharge that transportation costs represents for parents.

The orientation relatives receive from health system personnel influence the decision to accept IPT. Due to the lack of education and inadequate sensitization of the community [30], the rejection by caregivers, parents and/or guardians has been made evident as a barrier by other studies [42]. It can be improved by positive modelling of behavior.

This is one of the few studies in Latin America, and the first in the DR, to address a priority issue for the control of TB: barriers and facilitators of interventions for IPT administration in children under 5 years of age. Additionally, using quantitative and qualitative approaches allows to understand the real situation with more detail in this context, as it describes the experience of health system personnel involved in the intervention, at both administrative and assistance levels, as well as that of the relatives.

However, due to sample size limitations, these results must be interpreted with caution. Also, due to the collection of information from secondary sources and given that almost half of the records of patients with TB diagnoses did not have information on their contacts, it was not possible to establish the proportion of children under 5 years of age who were contacts of persons diagnosed with smear-positive TB in this area. Also, in some cases information was simply not available in the files. Records lacked information on TST results, dates of determination of resistance status of the index cases and ages were not registered by date of birth.

Implementation of person centred care is needed and should be part of the TCP’s policy to reduce TB disease burden. It is also necessary to do further research to address the sociocultural aspects that influence adequate IPT implementation, particularly those related with stigmatization. Development of tools and education protocols for those who are beginning to work in the TCP is needed to minimize data loss and thus be able to stop TB. In addition, it is important to consider the role of health system personnel as well as children with their families and social environment. This would provide information for the creation of educational and ludic strategies about TB prevention and control in educational centres in the Dominican territory, to improve intervention in this context. In fact, educational material was designed as a byproduct of this investigation and provided to the TB control program for use throughout the country. Overall, it is important to design and evaluate an intervention for IPT administration that is centered on the patient and his/her family, considers international guidelines, and includes socioeconomic aspects, such as alternatives to cover transportation and food costs.

## Conclusions

Incomplete information in program records and lack of implementation of TST for detection of the disease in children contributed to the low supply of IPT. Children who did receive IPT came from socially marginalized zones and lived in overcrowded conditions. These social conditions of children and their families; stigma by health system personnel, the family and the social surroundings result in omission of information. Lack of information in clinical records and follow-up forms, lack of coordination between public and private sectors and lack of coherence between national guidelines, were the main barriers identified.

Homecare of the person with TB and contacts, transfer of treatment to a healthcare centre near the child’s household, continuous availability of isoniazid, provision of information by health workers, and economic support for food and transportation, are some of the facilitators for the administration of IPT detected by this study. Most of these are aspects of a person centred approach.

## Data Availability

All data generated or analyzed during this study are included in this published article (and its Additional files).

## Abbreviations

BCG: Bacillum Calmette-Guerin
DAS: IV Area Directorate of Health
WHO: World Health Organization
TCP: Tuberculosis Control Programs
TST: Tuberculin Purified Protein Derivative
TB: Tuberculosis
IPT: Isoniazid Preventive Therapy
